# Human Gut Microbiota and Gastrointestinal Cancer

**DOI:** 10.1016/j.gpb.2017.06.002

**Published:** 2018-02-21

**Authors:** Changting Meng, Chunmei Bai, Thomas D. Brown, Leroy E. Hood, Qiang Tian

**Affiliations:** 1 Institute for Systems Biology, Seattle, WA 98109, USA; 2 Department of Oncology, Peking Union Medical College Hospital, Beijing 100730, China; 3 Swedish Cancer Institute, Seattle, WA 98104, USA; 4 P4 Medicine Institute, Seattle, WA 98109, USA

**Keywords:** Inflammation, Immune regulation, Microbial metabolites, Carcinogenesis, Traditional Chinese Medicine

## Abstract

Human gut microbiota play an essential role in both healthy and diseased states of humans. In the past decade, the interactions between microorganisms and tumors have attracted much attention in the efforts to understand various features of the complex microbial communities, as well as the possible mechanisms through which the microbiota are involved in cancer prevention, **carcinogenesis**, and anti-cancer therapy. A large number of studies have indicated that microbial dysbiosis contributes to cancer susceptibility via multiple pathways. Further studies have suggested that the microbiota and their associated metabolites are not only closely related to carcinogenesis by inducing **inflammation** and immune dysregulation, which lead to genetic instability, but also interfere with the pharmacodynamics of anticancer agents. In this article, we mainly reviewed the influence of gut microbiota on cancers in the gastrointestinal (GI) tract (including esophageal, gastric, colorectal, liver, and pancreatic cancers) and the regulation of microbiota by diet, prebiotics, probiotics, synbiotics, antibiotics, or the **Traditional Chinese Medicine**. We also proposed some new strategies in the prevention and treatment of GI cancers that could be explored in the future. We hope that this review could provide a comprehensive overview of the studies on the interactions between the gut microbiota and GI cancers, which are likely to yield translational opportunities to reduce cancer morbidity and mortality by improving prevention, diagnosis, and treatment.

## Introduction

Cancer has remained a serious concern in human society worldwide. Carcinogenesis is a well-known multi-factorial process, involving genetic and environmental perturbations. 15.4%–17.8% of cancers since 1990 were estimated to be related to infections, which accounted for 21.0%–26.3% and 5.0–9.0% of the cases in developing and developed countries, respectively [1], [2], [3], [4]. Nonetheless, among the 3.7 × 10^30^ microorganisms on the earth, only a few have been defined as carcinogenic agents by the International Agency for Research on Cancer (IARC). These include *Helicobacter pylori*, hepatitis B virus, hepatitis C virus, HIV type 1, human papillomavirus, Epstein-Barr virus, human herpesvirus type 8, human T-cell lymphotropic virus type 1, *Opisthorchis viverrini*, *Clonorchis sinensis*, and *Schistosoma haematobium* [4]*.* Although humans are colonized by trillions of microbes in general, only some individuals suffer from cancers. Therefore, the host, microbiota, and many other risk-driving factors are believed to be collectively responsible for the process of carcinogenesis [5].

The human gut is perhaps one of the most complex networks in the body and is colonized by trillions of microorganisms including bacteria, archaea, fungi, protists, and viruses, among which bacteria are the major inhabitants [6]. For decades, researchers have been trying to understand the complex relationships between the human microbiota and diseases. Mounting evidence has suggested that the gut microbiota are related to a variety of cancers, which may enlighten potential development of cancer therapies targeted at the gut microbiome [5], [7]. This review provides a comprehensive survey of the studies on the human gut microbiota and GI cancers, specifically esophageal, gastric, colorectal, liver, and pancreatic cancers.

During the last 30 years, gene-based and culture-independent methods for microbial profiling, *e.g.*, 16S rRNA sequencing, have made remarkable progress [8], [9] and have been used to differentiate and quantitatively evaluate various bacterial species as the method of choice [10]. High-throughput sequencing technologies, such as next-generation sequencing and random shotgun sequencing, as well as omics-based approaches, have enabled a more comprehensive examination of microbial communities without cultivation [11]. Specially, TruSeq, a synthetic long-read sequencing technology, allows researchers to assemble whole microbial genomes more completely [12]. Big data generated with these new sequencing technologies have been accurately analyzed using advanced computational strategies, such as genome assembly and gene-finding software, statistical modeling and simulations, and gene annotation tools. The flourishing advancements in computation and sequencing technologies have significantly promoted the development of the entire field of human microbiology.

It is now known that Actinobacteria, Firmicutes, Bacteroidetes, and Proteobacteria phyla are predominant in the human stomach, whereas Proteobacteria, Firmicutes, and Bacteroidetes phyla are frequently found in the colon tract [13]. Symbiotic gut microbiota have been characterized by high diversity, stability, resistance, and resilience, whereas dysbiotic gut microbiota exhibit low relative abundance as well as loss of commensalism and diversity.

Various studies have demonstrated that the carcinogenicity is mainly attributed to microbial dysbiosis (Table 1). (1) Chronic inflammation: Chronic inflammation has been verified as a driving cause of cancer. Inflammation promotes tumor progression and accelerates the invasion and metastasis. Inflammatory cytokines directly lead to DNA damage in the epithelium. Aberrant DNA methylation triggers inflammation-associated cancers [14]. Increased interleukin-1, 6, 10, and tumor necrosis factor-α (TNF-α) levels will initiate the process of cancer development, followed by three steps. These include (I) the activation of nuclear factor kappa-light-chain-enhancer of activated B cells (NF-κB), Wnt signaling and mitogen-activated protein kinases (MAPK) pathway, (II) the inhibition of apoptosis, and (III) a boost in oxidative stress [15]. IL-6 and IL-11 can sensitize signal transducer and activator of transcription 3 (STAT3), which exerts a significant impact on transforming epithelial cells [16]. β-catenin, forming complexes with adenomatous polyposis coli (APC), glycogen synthase kinase (GSK) 3β, and axin, can cause aberrations in the Wnt pathway in epithelial cells, thus activating proto-oncogenes encoding c-myc and cyclin D1 [17], [18]. The generation of inflammation-associated factors can also inactivate tumor-suppressor genes (*e.g.*, *P53* mutation), and activate oncogenes (*e.g.*, *KRAS* mutation) [19], [20]. (2) Immune regulation: Dysbiosis of the gut microbiota triggers a number of innate and adaptive immune responses involved in the tumor formation process [21], [22], [23]. The innate immune system can recognize the structural components of bacteria, such as flagellin, lipopolysaccharide (LPS), and peptidoglycan [23], [24]. Toll-like receptors (TLRs) play a critical role in the innate immune system given their ability in distinguishing microbial molecules from host molecules. Nod-like receptors (NLRs) also regulate the innate immune response, correspondingly modulating microbial composition and activating inflammasome-mediated dysbiosis. Additionally, T helper (T_h_) cells, T regulatory (Treg) cells, and B cells, which collectively secrete immunoglobulin A (IgA), participate in tumorigenesis through the adaptive immune system [22]. (3) Microbial metabolites: Lipoteichoic acid (LTA), secondary bile acids, and short chain fatty acids (SCFAs) have dual roles in carcinogenesis [25]. LTA specifically binds to cluster of differentiation 14 (CD14) or TLR2, causing excessive secretion of proinflammatory factors [26], [27]. Secondary bile acids activate G protein-coupled bile acid receptor 1 (GPBAR1), which increases intestinal cell proliferation [28], promotes DNA damage [29], and induces cellular senescence, leading to a senescence-associated secretory phenotype [30]. These aforementioned microbial metabolites advance malignant transformation. On the contrary, SCFAs are able to mediate immunoregulation through Tregs, therefore exhibiting anti-inflammatory and anti-carcinogenic effects [31], [32], [33].

**Table 1:** **GI cancerand microbiota**

Cancer	Condition	Microbes associated	Virulence or risk factor	Mechanisms	Refs.
Gastric cancer	*H. pylori* infection^*^	*H. pylori*	CagA	Immune responses and inflammation	[34], [35], [36], [37], [38], [39], [40]
				↑IFN-γ, TNF-α, IL-1, IL1β, IL-6, IL-7, IL-8, IL-10, and IL-18	
				↑Immune cells (lymphocytes, peripheral mononuclear cells, eosinophils, macrophages, neutrophils, mast cells, and dendritic cells)	
				↑Oncogenic signaling pathways (ERK/MAPK, PI3K/Akt, NFκB, Wnt/β-catenin, Ras, sonic Hedgehog, and STAT3)	
				↓Tumor suppressor pathways	
				↑p53 mutation	

			VacA	↑Autophagy	[41], [42], [43], [44], [45], [46], [47], [48], [49], [50], [51], [52], [53], [54], [55]
				↑MAP kinase and ERK1/2	
				↑VEGF, Wnt/β-catenin	
				↓PI3K/Akt, GSK3	
				↑Methylation of CpG islands, tumor-suppressor genes (*e.g.*, *TFF2*, *FOXD3*)	

	Non-*H. pylori*	↓Porphyromonas, Neisseria, the TM7 group, *Prevotella pallens*, and *Streptococcus sinensis*;↑ *Lactobacillus coleohominis*, *Klebsiella pneumoniae*, *Acinetobacter baumannii*			[60], [61], [62]

Esophageal cancer	GERD, Barrett’s esophagus	Enterobacteriaceae		Antibiotics and PPI alter the microbiome	[68], [69], [70]

	*H. pylori* infection^**^	*H. pylori*		Increase pH in gastric tract	[66]

	Early ESCC and ESD	Gram-positive bacteria (Proteobacteria, Firmicutes, Bacteroidetes, Actinobacteria, and Fusobacteria)	LPS	↑Immune responses, NF-κB (74)	[65], [71], [72], [73], [74], [75], [76]
				↑Inflammatory cytokines (IL1β, IL6, IL8, TNFα)	
				↑ iNOS, NO	

Colorectal cancer	High-fat diets	↑Sulfate-reducing bacteria (*Desulfovibrio vulgaris*)	LPS, LTA	Transform primary bile acids to secondary bile acids	[81]

				↓Cell death; ↑cellular immune response; ↑proinflammatory cytokine	[26], [78], [79], [80]

	Starches and dietary fiber^***^	Intestinal microbial fermentation (*Faecalibacterium prausnitzii* and *Eubacterium rectale*)	SCFAs (BA)	↓Pro-inflammatory mediators (iNOS, COX2, TNF-α, IL-1β, and IL-6)	[85]
				↓DNA methylation-mediated GPR109a silencing	[86], [87]
				↑p21 gene, c-fos and ERK1/2 phosphorylation	[88], [89]

	Some fruits and nuts^***^		Urolithins	Anti-inflammatory and anticancer effects by inhibiting Wnt signaling	[90], [91]

	Colorectal adenomas	↑Proteobacteria; ↓Bacteroidetes		Stimulating E-cadherin, β-catenin, NF-κB, and STAT3	[105], [106], [107]

	IBD	ETBF	*B. fragilis* toxin	↑TGFβ, TNFα, NF-κB, and ROS	[97]
				↑ E-cadherin, β-catenin, NF-κB, and STAT3	[99], [100]

			Fragilysin	↑IL-8, TGFβ, ENA-78, and GRO-α; ↑proliferation of colonic epithelial cell; ↑oncogene c-Myc	[101], [102]
	Adenomatous polyp, CRC	Fusobacterium	FadA	↑Expression of transcription factors, oncogenes, Wnt genes, and inflammatory genes	[108], [109], [110], [111]

	Other microbiota dysbiosis	*Escherichia coli*, *Shigella dysenteriae*, *Actinobacillus actinomycetemcomitans*, *Campylobacter* spp., *Helicobacter* spp., *Salmonella typhi*, and *H. ducreyi*	CDT, cytotoxic necrotizing factor 1, *B. fragilis* toxin, and colibactin	DNA Damage in colonic epithelial cells	[123], [124], [125], [126], [127], [128], [129], [130], [131], [132], [133], [134], [135]
				↑Unrepaired DNA pieces and BER	
				↑*PIK3CA* mutations	
				↑TGFβ, Wnt, and Notch promotes tumor progression and migration	
				↑ EMT	

Liver cancer	High-fat diet, obesity	Dysbiosis of gut microbiota	LPS, DCA	↑ DNA damage, SASP, inflammatory responses	[142], [143], [144], [145], [146], [147], [148], [149], [150], [151], [152], [153], [154], [155], [156], [157], [158], [159], [160], [161], [162], [163], [164], [165], [166], [167], [168], [169], [170]
				↑Intestinal permeability	
				↑NF-κB, Wnt/β-catenin, hepatocyte turnover, and oxidative injury	

	*H. pylori* infection	*H. pylori*	VacA, CagA, LPS	↑NF-κB, activator protein-1, and IL-8	[154], [155], [156]

	HCC	↑*Escherichia coli*	LPS		[147]

	CCA	↑Dietziaceae, Pseudomonadaceae, and Oxalobacteraceae			[148]

Pancreatic cancer	Microbial infection	*H. pylori*	Ammonia, LPS	↑*KRas* gene mutation, STAT3, NF-κB, MAPK signal pathways	[174], [175], [176], [177], [181], [182], [183], [184], [185], [186], [187], [188]
				↑Inflammasomes (NF-κB, activator protein-1, IL-8)	
				↑Anti-apoptotic and pro-proliferative proteins (Bcl-XL, Mcl-1, survivin, c-Myc, and cyclin D1)	
				↑Immune responses	[186], [190], [191], [192], [193]

		*Pseudomonas aeruginosa*		Natural ligand for T2R38	[196]

		*Fusobacterium* species^****^		Independent negative prognostic biomarker	[197]

*Note*: ALD, alcoholic liver disease; BER, base excision repair; GERD, Gastroesophageal reflux disease; IBD, Inflammatory bowel disease; NAFLD, non-alcoholic fatty liver disease; CCA, cholangiocarcinoma; CDT, cytolethal distending toxin; CRC, colorectal cancer; DCA, deoxycholic acid; EMT, Epithelial-to-mesenchymal transition; ETBF, Enterotoxigenic *Bacteroides fragilis*; GRO-α, growth related oncogene-α; HCC, hepatocellular carcinoma; FadA, *Fusobacterium* adhesion A; SASP, senescence-associated secretory phenotype; LPS, lipopolysaccharide; PPI, proton pump inhibitor; NO; nitric oxide; iNOS; inducible nitric oxide synthase; LTA, lipoteichoic acid; SCFA, short-chain fatty acid; BA, butyric acid. ^*^, class I risk factor; ^**^, decreased risk factor; ^***^, anti-tumorigenic; ↑, increased, upregulated, activated; ↓, decreased, downregulated, inhibited.

## Gut microbiota and gastric cancer

### *H* pylori

Gastric cancer is considered as an inflammation-associated cancer. Known as a Class I risk factor, infection by *H. pylori* can stimulate immune responses and inflammation, regulate many signaling pathways, and induce achlorhydria, epithelial atrophy, and dysplasia. Therefore, effective eradication of *H. pylori* could prevent gastric cancer [34].

Oncoproteins cytotoxin-associated gene A (CagA) and vacuolating toxin A (VacA) are critical virulence factors of *H. pylori* [35]. Cag^+^ strain infections present a highly increased risk of gastric cancers [36], [37]. Elevated accumulation of inflammatory cytokines is found in the stomach of *H. pylori*-infected individuals, which include interferon-γ, TNF-α, IL-1, IL1β, IL-6, IL-7, IL-8, IL-10, and IL-18. Consequently, diverse types of immune cells are stimulated, encompassing lymphocytes, peripheral mononuclear cells, eosinophils, macrophages, neutrophils, mast cells, and dendritic cells. The activity of oncogenic pathways containing ERK/MAPK, PI3K/Akt, NF-κB, Wnt/β-catenin, Ras, sonic hedgehog, as well as STAT3 is upregulated with the infection of Cag^+^ *H. pylori* strains. Conversely, tumor suppressor pathways are inactivated with induced *P53* mutations [38], [39], [40].

VacA can cause cell vacuolation [41], [42], [43] and induce autophagy within human-derived gastric epithelial cells [44], [45], by acting directly on mitochondria [46], [47], [48], upregulating MAP kinase and ERK1/2 expression [49], activating vascular endothelial growth factor [50], [51], upregulating Wnt/β-catenin signaling pathway which is essential for cell growth and differentiation [52], and inhibiting GSK3 via the PI3K/Akt signaling pathway [53].

Furthermore, *H. pylori* infection can cause methylations on CpG islands of E-cadherin [54] and tumor-suppressor genes, including those encoding the trefoil factor 2 (*TFF2*) and a forkhead box transcriptional regulator (*FOXD3*), resulting in the significantly increased risk of adenocarcinoma in the stomach [55].

### Non-*H. pylori* microbiota

Current sequencing technologies allow researchers to dive deeply into the complexity of gut microbiota, which may be influenced by multiple factors [56]. Microbial community in *H. pylori-*positive individuals is characterized by an increase in the counts of Proteobacteria, Spirochaetes, and Acidobacteria, as well as a decrease in the counts of Actinobacteria, Bacteroidetes, and Firmicutes [57]. Conversely, *H. pylori*-negative individuals carry more abundant phyla of Firmicutes, Bacteroidetes, and Actinobacteria [58]. Microbial dysbiosis is also associated with gastric carcinogenesis [59]. Using quantitative PCR, it has been shown that gastric cancer patients bear a much diversified composition of microbiota, exemplified by the reduction of *Porphyromonas*, *Neisseria*, the TM7 group, *Prevotella pallens*, *Streptococcus sinensis*, and simultaneous enrichment of *Lactobacillus coleohominis*, *Klebsiella pneumoniae*, *Acinetobacter baumannii*, and Lachnospiraceae [60], [61], [62]. Pathogenic components derived from *Helicobacter* species other than *H. pylori*, such as the outer membrane proteins phospholipase C-gamma 2, BAK protein, and nickel-binding proteins, assist microbes with colonization in the mucosal layer of the gastric tract and then promote the process of gastritis, ultimately enhancing the possibility of tumorigenesis in the stomach [56]. To precisely elucidate the correlations between the microbial dynamics and pathogenesis of gastric cancer, further functional and mechanistic studies are needed.

## Gut microbiota and esophageal cancer

Esophageal cancer (EC) is subdivided histologically into two major groups: esophageal squamous cell carcinoma (ESCC) and esophageal adenocarcinoma (EAC). It has been reported that the upper aerodigestive tract carcinomas are closely associated with common potential risk factors, such as infections from the human papilloma [61], [63], [64] and Epstein-Barr viruses, although the pathogenic mechanism(s) remain controversial [65]. In addition to viruses, bacterial infections also contribute to the formation of esophageal malignant neoplasms.

### *H* *pylori* constitutes a decreased risk of EC

In the last 20 years, the incidence rate of EAC has shown a tendency of reduction in the general population infected with *H. pylori*, especially in Eastern populations. In the meantime, the incidence of ESCC has also diminished [66]. Gastroesophageal reflux disease (GERD) is a leading cause of Barrett's esophagus, a premalignant condition of EAC [67]. By inhibiting parietal cell function and/or inducing the development of atrophic gastritis, chronic *H. pylori* infections can restrain parietal cells from secreting hydrochloric acid, thus increasing the pH in the gastric tract and eventually leading to a reduction of EAC. There is a higher relative abundance of Enterobacteriaceae in the stomach of patients with oesophagitis and Barrett’s esophagus compared to normal populations. It has been suggested that antibiotics may alter the microbiome in the esophagus of patients with GERD [68]. Gut microbiota colonized in the esophagus and stomach are notably altered by treatment with proton pump inhibitors (PPIs). However, it is not conclusive whether the changes caused by PPIs are beneficial or not [69]. The latest systematic review and meta-analysis show that PPIs do not decrease the development of dysplasia and Barrett’s esophagus-related EAC [70].

### Other gut microbiota and EC

The esophagus is traditionally considered as a microbe-free site, with limited microbial passengers coming from swallowing and gastroesophageal reflux. By applying 16S rRNA sequencing technology, some specific microbes were found to populate the esophageal mucosa, including Firmicutes, Bacteroidetes, Proteobacteria, Actinobacteria, and Fusobacteria phyla. Moreover, distinct microbial communities were found in esophagus of individuals with ESCC (stage I–II) and esophageal squamous dysplasia (ESD), compared to normal esophagus [71]. Consistent with the normal gastric mucosa microbiota, the most common phyla in the samples of early ESCC and ESD are Proteobacteria, Firmicutes, and Bacteroidetes [71], which are involved in the tumorigenic process in the esophagus when esophageal microbiota are dysbiotic [65], [72]. It has been found that the human distal esophagus has its own characteristic microbiota. Gram-positive bacteria, including Firmicutes and *Streptococcus* were dominant in the normal esophagus, while gram-negative anaerobes/microaerophiles, such as Bacteroidetes, Proteobacteria, Fusobacteria, and Spirochaetes, were mainly associated with esophagitis and Barrett's esophagus [73]. LPS, an important component of gram-negative bacterial cell wall, participates in the oncogenic process through multiple mechanisms. These include activating innate immune responses that lead to NF-κB activation [74], promoting the release of inflammation-associated mediators including IL1β, IL6, IL8, and TNFα [75], raising the levels of inducible nitric oxide synthase (iNOS) and nitric oxide (NO), increasing the risk of reflux through relaxing lower esophageal sphincter, and delaying gastric emptying [76].

## Gut microbiota and colorectal cancer

The gut microbiome in the large intestinal tract is the most complicated community in the human body. The bacterial population primarily comprises gram-positive Firmicutes, and gram-negative Bacteroidetes and Proteobacteria.

### Diet, microbial metabolites, and colorectal cancer

Various factors contribute to colorectal cancer (CRC) and diet is a well-known and important environmental factor associated with CRC. Many different gut microbiota metabolites have either tumorigenic or anti-tumorigenic characteristics.

The subunits of the LPS receptor expressed on colonocytes inhibit cell death, activate cellular immune response via TLR2, and then stimulate downstream proinflammatory cytokine signaling, leading to tumorigenesis [26], [77], [78]. LTA, an element arising from the cell wall of gram-positive bacteria, is regarded as the counterpart of LPS, the component of gram-negative bacterial cell wall [79]. High-fat diets boost the relative abundance of sulfate-reducing bacteria, such as *Desulfovibrio vulgaris*, which transforms primary bile acids to secondary bile acids such as lithocholic acid and deoxycholic acid, is potentially tumorigenic [80]. Conversely, butyric acid (BA), an important short-chain fatty acid (SCFA) that is generated from fermentable fibers in diets by colonic bacteria, has been shown to be anti-tumorigenic [81]. The most important butyrate-producing microbial groups that are involved in the process of fermentation are *Faecalibacterium prausnitzii* and *Eubacterium rectale* [82]. BA is utilized by the mitochondria in colonocytes, which helps to maintain a healthy energy balance and benefit colonic epithelial cell proliferation [83]. GPR109a, a receptor of SCFAs expressed on immune cells, primarily activates the ligands of BA, then inhibits inflammatory cytokines, thus suppressing the process of inflammation [84]. The host immune response fights back DNA methylation-mediated GPR109a silencing through IFNγ, therefore encouraging anti-carcinogenic effects accordingly [85], [86]. In addition, BA exerts various chemopreventive effects by inducing *P21* gene expression, inhibiting the activator protein-1 (AP-1) signaling pathway, and increasing the phosphorylation of c-Fos and ERK1/2 [87], [88]. Additionally, urolithins such as urolithin A are intestinal microflora metabolites of fruits and nuts with plenty of ellagic acid. They have been reported to inhibit Wnt signaling and show benefits against cancer [89], [90].

### Chronic inflammation and CRC

Chronic inflammation produces considerable inflammatory mediators, such as TNFα, IL6, IL1b, and other cytokines, which activate NF-κB, leading to colon carcinogenesis [91]. Inflammatory bowel diseases (IBDs) link to a higher risk of CRC. For instance, patients with pancolitis have a more serious risk to develop cancer compared to patients with limited colitis [92]. Gut microbiota of IBD patients have less diversity and dysbiosis, characterized with lower abundance of Firmicutes and Bacteroidetes, compared with healthy subjects [93]. Enterotoxigenic *Bacteroides fragilis* (ETBF) exhibits a significant correlation with the presence of active IBD [94], [95]. Both IBD and CRC share a common process with an increase in the levels of transforming growth factor-beta (TGF-β), TNFα, NF-κB, ROS, and other signaling molecules, leading to microbial dysbiosis in the intestinal tract [96]. It has been demonstrated that patients with CRC accompanied by IBD have a worse prognosis than those without IBD only [97].

Toxins secreted by *B.fragilis* can result in tumorigenesis in the large intestine by stimulating E-cadherin, β-catenin, NF-κB, and STAT3 [98], [99]. For instance, fragilysin, an enterotoxin secreted by *B.fragilis*, stimulates expression of IL-8, TGFβ, C5a, leukotriene 4 (LTB4), and growth related oncogene-α (GRO-α), resulting in an inflammatory environment [100]. Moreover, fragilysin induces proliferation of colonic epithelial cells and the expression of the oncogene *c-myc* [101]. Adenomatous polyps or adenomas are considered premalignant for CRC. The diversity, relative abundance, and distribution of the gut microbiota in adenoma populations are significantly different from those in healthy populations [102]. Patients with colorectal adenomas harbor significantly more Proteobacteria as well as less Bacteroidetes compared with healthy controls. The bacteria adherent to the colorectal mucus layer form a particular biofilm and intervene in the formation of adenomatous polyps in the colon [103]. Dysbiosis of the gut microbiota can thus promote the process of tumor formation in the large intestine tract [104], [105], [106].

*Fusobacterium* adhesin A (FadA), a cell surface virulence factor expressed by *Fusobacterium*, is frequently detected in patients with adenomatous polyp or CRC. FadA interacts with E-cadherin on the endothelium and modulates the E-cadherin/β-catenin pathway, resulting in an increased expression of transcription factors, oncogenes, and inflammatory genes. It also promotes *Fusobacterium* to adhere to and invade E-cadherin-expressing cells, thereby, directly influencing epithelial cell proliferation and growth [107], [108], [109]. A recent report has indicated that the overall abundance of *Fusobacterium* in CRC tissues is over 400 times higher than that in the adjacent normal tissues [110]. Therefore, FadA may be a potential biomarker for the diagnosis and therapy of CRC.

### Immune regulation and CRC

Upon interacting with microorganisms and their gene products, dendritic cells become activated, switching on the gut immune response [111]. The host innate immune system can recognize microbial molecules, including LPS, flagellin, peptidoglycans, and other microbe-associated molecular patterns (MAMPs). The activation of pattern recognition receptors, for instance, NLRs and TLRs, regulates inflammatory pathways and the proliferation of multiple cell types [112], [113], [114]. NLR-mediated inflammasome activation and enhanced TLR2 expression play a protective role in maintaining the complete structure and function of colonic epithelium by suppressing the inflammatory environment [115], [116], [117].

TLRs are confirmed to recognize diverse molecules from microbes and promote tumorigenesis [118]. Protein levels of TLR2, 4, 7, 8, and 9 are increased in CRC tumor tissues compared to those in the healthy surrounding tissues [119]. Overexpression of TLR4 results in the activation of β-catenin and increased colitis-associated cancer development, whereas the inhibition of TLR4 expression is shown to protect against CRC [118].

In a meta-analysis of microarray studies, a significant difference in the NLR signaling pathways between tumor tissue and non-tumor tissue of patients with CRC has been observed [114], [120]. The nucleotide-binding oligomerization domain-containing protein 1 (Nod1), an innate immune receptor and a NLR, recognizes microbial molecules, then initiates immune responses, and inhibits the tumorigenic process. In contrast, Nod1 deficiency increases intestinal permeability, leading to colitis-associated cancers [121].

### Gut microbiota dysbiosis and genetic instability

The diversity and abundance of beneficial commensals could be minimized, if gut microbiota remain at the dysbiotic state. Once the disturbed microbes overgrow, they give rise to accumulating exotoxins and endotoxins, such as cytolethal distending toxin and colibactin from *Escherichia coli*, cytolethal distending toxin from *Shigella dysenteriae*, *B. fragilis* toxin from *B. fragilis*, extracellular superoxide, and hydrogen peroxide from *Enterococcus faecalis*, etc. These bacterial toxins are able to directly or indirectly induce DNA damage, genomic instability, tumorigenesis, and the invasion of adenocarcinomas [122], [123], [124], [125], [126], [127], [128], [129], [130]. Additionally, dysbiosis results in increased the exposure of colonic epithelial cells to carcinogens [131]. The accumulation of unrepaired DNA and base excision repair (BER) intermediates leads to genomic instability and ultimately carcinogenesis [132], [133]. Furthermore, microbial dysbiosis can dysregulate the immune response and increase inflammation, resulting in *PIK3CA* mutations, which may accelerate the initiation and/or growth of rectal cancers [134].

### Gut microbe and epithelial-to-mesenchymal transition

Microbes induce epithelial-to-mesenchymal transitions (EMT) through various signaling pathways, such as TGFβ, Wnt, and Notch, which work together with the transcription factors (TFs) Slug, SNAIL, Twist, ZEB1, and ZEB2 to suppress E-cadherin, leading to tumor invasion, metastasis, and acquired drug resistance [135], [136], [137]. It is worth noting that the cells undergoing EMT are claimed to obtain stem cell-like properties and thus constitute a cancer stem cell (CSC) population [138], [139].

## Gut microbiota and liver cancer

Although liver is generally considered sterile, the hepatic environment is greatly influenced by the pathogens or metabolites produced by the microbiota in the GI tract through the hepatic portal venous system [140]. Liver exerts an essential effect on the host microbial community by filtering the blood stream as well as metabolizing and neutralizing toxins derived from intestinal microbes. Gut microbial dysbiosis contributes to hepatocarcinogenesis because the microbiota and microbial metabolites are detected by liver resident immune cells and are able to modify hepatic metabolism [140].

Hepatocellular carcinoma (HCC) and cholangiocarcinoma (CCA) are the most common histological types of liver cancer. Alcoholic liver disease (ALD), non-alcoholic fatty liver disease (NAFLD), as well as infections with foodborne contaminant aflatoxin B1 (AFB1), hepatitis B or C virus [141] are considered as the major risk factors for HCC [142], [143]. Of note, dysbiosis of the gut microbiota is one of the key inducers for non-alcoholic fatty liver disease [144], [145]. The abundance of *E. coli* in feces from patients with HCC is much higher than that in feces from healthy controls [146], while *Dietziaceae*, *Pseudomonadaceae*, and *Oxalobacteraceae* are more abundant in the bile duct samples from patients with CCA than samples from non-CCA individuals. It has been hypothesized that excessive microbial growth in the gut may promote liver cancer development [147], which needs to be further explored.

### *H* *pylori* and liver cancer

*H. pylori* generally inhabits the human stomach [148], [149]. However, *H. pylori* from the gut can reach the liver tissue [150] through the blood stream of the portal vein [151], [152] after surviving phagocytic elimination, or by reverse migration via the duodenum. VacA and CagA produced by *H. pylori* have been found in liver tissues with HCC [153], [154]. It has been shown that LPS from *H. pylori* directly promotes the growth and migration of liver cancer by increasing the levels of IL-8 and TGF-β1 [155].

As a member of the Helicobacteraceae family, *H. hepaticus* causes the development of HCC by activating the NF-κB and Wnt signaling pathways, hepatocyte turnover, and oxidative stress [156]. Additionally, some *Helicobacter* species, such as *H. pylori*, *H. bilis*, *H. hepaticus*, and *H. ganmani*, are specifically related to CCA, but not non-tumor diseases in the bile duct [157].

### Gut microbial metabolites and liver cancer

Microbial metabolites disturb the metabolic pathways and immune response in the liver. TLR4 recognizes LPS coming from bacteria and activates Kupffer cells through LPS-induced TNF-β and IL-6 [158]. It can also stimulate stellate cells through growth factors such as epiregulin [159], and initiate various inflammatory and oncogenic pathways [160]. The LPS-TLR4 pathway promotes HCC, whereas removal of LPS or genetic inactivation of TLR4 could decrease HCC development [159]. However, whether the intestinal microbiota and TLR4 contribute to HCC initiation remains controversial [159], [161].

Cholic acid and chenodeoxycholic acid are the major primary bile acids produced by the liver. They cause DNA damage by increasing the production of reactive oxygen species (ROS), thus inducing the development of liver cancers [162]. In addition, bile acids are also confirmed to regulate the gut microbiome. Decreased quantities of bile acid result in the overgrowth of gut microbiota and accelerate inflammation [163]. The enterohepatic circulation of deoxycholic acid (DCA) produced by *Clostridium* causes DNA damage and provokes a senescence-associated secretory phenotype (SASP) in hepatic stellate cells. This process involves numerous inflammatory cytokines and growth factors, thereby contributing to inflammatory and obesity-associated HCC transitions [164], [165], [166]. DCA and lithocholic acid are shown to directly promote cancer through DNA damage [29].

### Gut microbiota, obesity, and liver cancer

Obesity increases the likelihood of various cancers, such as liver cancer [167], [168], and causes microbial dysbiosis. Under the obesity condition, tight junctions of gut epithelium get degraded due to chronic inflammation. As a result, there is an increase in intestinal permeability, as well as bacterial counts and the levels of metabolites translocated from the gut epithelium into circulation because of the chronic inflammation [166]. IL-6 and plasminogen activator inhibitor-1 induced in obesity also lead to inflammatory responses and tumorigenesis [169]. In addition, the number of gram-positive bacteria as well as the serum level of DCA are increased in mice put on a high-fat diet, indicating that the DCA-SASP axis plays a critical role in the progression of obesity-associated liver cancer [164].

## Gut microbiota and pancreatic cancer

Pancreas is an extragastric digestive organ. Pancreatic ductal adenocarcinoma (PDAC), one of the most deadly cancers globally, is the most common type of pancreatic cancer. Accumulating studies have demonstrated that gut microbiota might influence pancreatic carcinogenesis [170], [171] by promoting inflammation, activating the immune response, and perpetuating cancer-associated inflammation [172].

### *H* *pylori* and pancreatic cancer

Risk factors for pancreatic adenocarcinoma include age, cigarette smoking, obesity, chronic pancreatitis, and diabetes. A review of hundreds of meta-analyses on pancreatic cancer revealed that *H. pylori* infection is another considerable risk factor for PDAC [173]. Besides PDAC [174], [175], [176], *H. pylori* is also involved in the acute and chronic pancreatitis [177], [178], [179], as well as autoimmune pancreatitis [180].

Many pathogenic components derived from *H. pylori*, including ammonia and LPS, as well as large quantities of resulting inflammatory cytokines, damage the pancreas [181]. *H. pylori* infections activate both NF-κB and AP-1, leading to dysregulation of cellular processes. Increased IL-8 levels accelerate inflammation, eventually resulting in pancreatic carcinogenesis [182]. *KRAS* performs an essential function in normal tissue signaling, while *KRAS* gene mutations are present in over 90% of the cases of pancreatic adenocarcinoma [183]. LPS from *H. pylori* is confirmed to hyperstimulate mutations of *KRAS* genes and initiate the process of pancreatic carcinogenesis [184], [185]. In addition, persistent STAT3 activation by *H. pylori* infections can promote pancreatic cancer progression by upregulating the expression of anti-apoptotic and pro-proliferative proteins, including Bcl-xL, MCL-1, survivin, c-myc, and cyclin D1 [173], [186], [187].

### Inflammation and immune response in pancreatic cancer

Microbes incur mild and sustained immune responses and inflammatory reactions, resulting in the formation of pancreatic cancer [188]. Many studies have been performed to explore the possible mechanisms. TLRs expressed on various immune cells enable the immune cells to recognize both numerous microbe-associated molecular patterns (MAMPs) and non-infectious inflammatory damage-associated molecular patterns (DAMPs), then activate the NF-κB and MAPK signaling pathways [185], [189]. Consequently, these processes initiate and perpetuate pancreatitis, and finally promote the progression of pancreatic cancer [190], [191], [192].

NLRs are cytoplasmic pattern recognition receptors (PRRs) that are involved in the activation of NF-κB and the formation of inflammasomes. P38 mitogen-activated protein kinases (P38 MAPKs) are responsive to cytokines, and are involved in cell differentiation, apoptosis, and autophagy, thereby accelerating the process of PDCA. Thus, P38 inhibitors are possible therapeutic agents for pancreatic cancer [193].

Taste receptor 2 member 38 (T2R38) is a bitter taste receptor. Interestingly, T2R38 is expressed not only in oral cells but also in pancreatic cancer cells. *Pseudomonas aeruginosa* is a unique ligand for T2R38 that is stated to activate T2R38, induce multi-drug resistance protein 1 (ABCB1), and get involved in cancer invasion and metastasis [194]. Additionally, *Fusobacterium* species exist in 8.8% of pancreatic cancer tissues. It is of note that the status of *Fusobacterium* species is an independent negative prognostic biomarker of pancreatic cancer [195].

## Future directions

Gut microbiota are closely related to GI cancers. Prebiotics, probiotics, synbiotics, and some specific antibiotics are often applied to build up a healthy gut. Quite a few recent studies have shown that gut microbiota affect the efficacy of antitumor treatments, including chemotherapy and immunotherapy.

### Prebiotics

The World Health Organization (WHO) defines “prebiotics” as “a non-viable food component that confers health benefit(s) on the host associated with modulation of the microbiota” [196]. A healthy diet, with increased consumption of plant foods and limited intake of meat, will be helpful to set up a healthy gut microbiota [197].

Dietary flaxseeds benefit the colonic microenvironment and reduce the susceptibility to gut-related diseases [198]. Inulin diets significantly decrease the pH of the cecal content, the concentration of phenol, p-cresol, and indole in the colon tract, inhibit the activity of microbial enzymes, including β-glucuronidase, azoreductase, and nitroreductase, and decrease the possibility of colonic precancerous lesions [199]. Avenanthramide-C (2c), an avenanthramide particularly found in oats, is extensively metabolized by gut bacteria and exerts an anti-inflammatory effect [200]. Urolithins are gut microflora metabolites of ellagitannins and ellagic acid. Intake of pomegranate extract significantly increases the quantities of ellagic acid and urolithins in the CRC tissues [201]. The hops plant, *Humulus lupulus* L., is a primary agent in beer containing prenyl flavonoids with weak estrogenic effect. Prenyl flavonoids are augmented by the gut microbiota, exerting anticancer effects on CRC models [202]. Agaro-oligosaccharides from seaweed show a positive effect on high-fat diet-induced gut dysbiosis and CRC development by altering the amount of SCFAs, bile acid, and phospholipids [203]. Nutmeg exhibits antimicrobial activity by decreasing IL-6 levels and normalizing dysregulated lipid metabolism [204]. Fermentation of nuts results in higher concentrations of SCFA, and the formation of vaccenic acid, a conjugated linoleic acid, which could be a potential chemopreventive metabolite [205]. Polyphenols subjected to microbial metabolism have both anti-carcinogenic and anti-mutagenic effect to prevent colon cancer [206]. Eicosapentaenoic acid-free fatty acid, an omega-3 fatty acid, effectively inhibits the process of inflammation as well as the formation of polyps and colitis-associated cancers [207]. Both the COLON study [208] and a RCT study [209] revealed the beneficial effects of polydextrose on gut microbiota and in the prevention of CRC. Another recent study has shown that although the intake of palm dates did not significantly change the relative abundance of gut microbiota or SCFAs, it can significantly increase bowel movements and stool frequency, while significantly reducing the stool ammonia concentration and the genotoxicity in human fecal water [210].

### Probiotics

Probiotics are defined by the Food and Drug Administration (FDA) and WHO as “live microorganisms, which when administered in adequate amounts, confer a health benefit on the host.” A number of studies have claimed the benefits of probiotics on the suppression of CRC, notably through participating in the innate immune system and apoptosis, decreasing oxidative stress, and improving the community of gut microbiota [211], [212], [213].

*Lactobacillus* species are the most commonly used probiotics in clinical trials because of a reduction in the abundance of *Enterobacter* and the regulation of immune response in gut of patients with CRC, whereas *Bifidobacterium longum* administration has no such effect [214]. *Lactobacillus salivarius* REN administration can effectively suppress the development of CRC in 1,2-dimethylhydrazine-induced experimental animals. It has been shown that the injection with this potent carcinogen remarkably altered the microbial community by increasing the number of *Ruminococcus* species (sp.) and Clostridiales bacteria, while decreasing the number of *Prevotella* sp*.* Furthermore, *Ren* intake promotes the rehabilitation of gut microbiota, suggesting that *Ren* may potentially be beneficial for the prevention of colon cancer [215]. A probiotic cocktail, comprising *Lactobacillus acidophilus*, *Bifidobacteria bifidum*, and *Bifidobacteria infantum* (LBB), enriched with oligofructose and maltodextrin, decreases the counts of the species of *Pseudomonas*, *Congregibacter*, *Clostridium*, *Escherichia*, *Helicobacter*, while increasing the counts of *Lactobacillus* in CRC [216]. Probiotic Prohep (a mixture of *Lactobacillus rhamnosus* GG [LGG], *E. coli* Nissle 1917 [EcN], and heat inactivated VSL#3 (probiotic medical food [1:1:1]) decreases the growth of HCC significantly by inhibiting angiogenesis and inflammation. It has been shown that the population of gut microbiota shifts to specific bacteria, such as *Prevotella* and *Oscillibacter*, creating favorable anti-inflammatory products. T_h_17 cells are pro-inflammatory T_h_ cells, which are able to produce interleukin 17 (IL-17) as an angiogenic factor. Prohep administration helps downregulate the T_h_17 frequency and the production of IL-1, inhibits the angiogenesis, and promotes the differentiation of anti-inflammatory Treg cells in the GI tract [217].

Furthermore, the conventional treatment of *H. pylori* infection with amoxicillin, clarithromycin, and PPIs can alter the indigenous gut microbiota to cause a long-term impact [218]. In a randomized controlled trial comparing the conventional treatment group to the combination treatment group with probiotics, the gut microbial community in the conventional treatment group is changed more significantly, with a greater proportion of drug-resistant bacteria. It has been pointed out that probiotic administration would help the gut microbiota fight back the perturbation induced by the treatment of *H. pylori* infection [219].

### Synbiotics

Synbiotics are combinations of prebiotics and probiotics [196]. A previous study has demonstrated that synbiotic supplementation during neoadjuvant chemotherapy for EC improves the gut microbial community and reduces the side effects caused by chemotherapeutic agents [220]. Since alterations in the gastric microbiome contribute to the increased incidence of esophageal adenocarcinomas, particularly those that arise within the gastroesophageal junction, the esophageal microbiome may be manipulated with antibiotics, probiotics, or inhibitors of specific host cell pathways to prevent disorders at this site [221].

### Antibiotics

The incidence and severity of colitis-associated cancer are reduced by administering antibiotics [222], [223]. Antibiotic administration during the primary inflammation stage can inhibit the initiation of carcinogenesis in an animal colonic cancer model [224].

As mentioned earlier, ETBF promotes the development of IBD as well as IL-17A-dependent CRC [225]. An ETBF-clearance mouse model was established by cefoxitin administration. It is found that expression of the mucosal IL-17A was inhibited with cefoxitin treatment. The ETBF clearance prohibits colon adenoma formation and IL-17A-dependent tumorigenesis. However, the effects of antibiotics are two-sided. Antibiotic exposure may induce cancers as well. A nested case-control investigation has demonstrated is a link between the exposure of penicillin and high risks of esophageal, gastric, and pancreatic cancers [226]. Another recent nested case-control study on liver cancer has also shown a trend of increased risk of liver cancer in cases having antibiotic therapy, compared to the cases without antibiotic therapy. However, it is uncertain whether the dose of antibiotics is correlated to the risk of liver cancer [227].

### COX2-inhibitor

Celecoxib is a selective COX2 inhibitor that alters the gastrointestinal microbial community as well as downregulates the polyp load in the gut. A recent study has found that celecoxib treatment significantly reduces the burden of polyps in a mouse model. In addition, the gut microbial community is characterized by decreased populations of *Lactobacillaceae* and *Bifidobacteriaceae* species, and an increased population of *Coriobacteriaceae* species. A metabolomics analysis shows that celecoxib treatment reduces the formation of pathogenic microbial products and thus inhibits cell proliferation [228].

### Pharmacodynamics of anticancer agents

#### Irinotecan

Irinotecan is one of the main chemotherapeutic agents for CRC patients. Gut microbiota mediate the toxicity of irinotecan (CPT-11) chemotherapy that can induce the loss of intestinal barrier function [229]. The counts of cecal *Clostridium* cluster XI, *Enterobacteriaceae*, pathogenic *E. coli*, and *Clostridium difficile* increased after CPT-11 administration. Glutamine treatment can induce temporary alternations of the gut microbiota and reduce the intestinal toxicity of CPT-11 [230].

#### 5-Fluorouracil

5-fluorouracil (5-FU) is an important chemotherapeutic agent for CRC treatment. However, usage of 5-FU is limited by chemoresistance. In 5-FU-resistant CRC cells, *Lactobacillus plantarum* supernatant (LPSN) inhibits the expression of particular biomarkers of cancer stem cells, promotes cell death and apoptosis, and selectively inactivates the Wnt/β-catenin signaling pathway, thus enhancing 5-FU efficacy, and reversing the development of resistance to anticancer drugs. This implies that probiotic substances could be useful therapeutic alternatives as biotherapeutics for chemoresistant CRCs [117]. Urolithin A, a gut microbial metabolite from diets containing ellagic acid, targets the colonic mucosa of patients with CRC, showing the capacity to counteract inflammation and prevent cancers. When co-treated with supplementary urolithin A, the IC50 values of 5-FU and 5′DFUR also decreased, indicating lower drug doses would be needed, thus reducing the side effects of hemotherapy [231].

#### Anticancer immunotherapy

Microbes can enhance the therapeutic effect of cancer immunotherapy [232]. A previous study has shown that the anti-cancer efficacy and immunostimulatory effect of cytotoxic T-lymphocyte-associated protein 4 (CTLA-4) blockade, an immune checkpoint blockade, rely upon different *Bacteroides* species, such as the *Bacteroides thetaiotaomicron* and *B. fragilis* [233]. The efficacy of programmed cell death protein 1 ligand 1 (PD-L1) inhibitors, another important immune-oncology therapy, is significantly augmented by *Bifidobacterium* administration. Moreover, tumor progression is almost prohibited after the treatment [234].

### Traditional Chinese Medicine

#### Baicalin and baicalein

Baicalin, a flavone glycoside isolated from the root of *Scutellaria baicalensis*, which is one of the commonly used herbs in the Traditional Chinese Medicine, slightly inhibits cell proliferation and induces apoptosis. Baicalein, a flavonoid isolated from *S. baicalensis*, has anti-inflammatory effects and strongly inhibits cell proliferation *in vitro*, particularly on CRC cells lines. It functions by arresting the cell cycle at the S phase, activating caspase 3 and caspase 9, thereby inducing apoptosis [235].

#### Curcumin

Curcumin, which is produced by the roots of the *Curcuma longa* plant, has potential anti-carcinogenesis properties by maintaining the diversity of gut microbiota. It has also been demonstrated to be an anti-inflammatory, anti-oxidative, and anti-proliferative agent [236]. However, numerous clinical studies assessing the efficacy of curcumin in cancer treatment has been inconclusive [237].

#### PHY906

PHY906 (a Chinese herbal medicine) can restore the gut epithelium through stimulating the regeneration of intestinal stem or progenitor cells upon transformation by bacterial β-glucuronidase, which is highly expressed by the gut microbiota. It was reported that PHY906 administration in advanced CRC patients reduces the GI toxicity of irinotecan and exerts an anti-tumor effect [238], [239], [240], [241], [242], [243].

### Prognostic biomarkers

*Fusobacterium nucleatum* may accelerate cancer progression and inhibit T cell-mediated immune responses in CRC. In a cohort consisting of 1069 CRC cases, the abundance of *F. nucleatum* was found to be related to high microsatellite instability and thus was independent of the *BRAF* mutation status. The higher quantity of *F. nucleatum* DNA in the tumor tissue was proportional to worse prognosis. Therefore, this may serve as a potential prognostic biomarker for colorectal cancers [244].

## Concluding remarks

Mechanistic studies trying to understand how gut microbes regulate body health and cancers are still at the early stage, revealing primarily a correlation rather than a causal relationship. However, people have realized that gut microbiota are closely and functionally related to the humans and play an important and unique role in human health and disease. People have begun to take bold efforts, trying to regulate gut microbes. The aims are multifaceted, ranging from regulating human metabolism, immune and inflammatory response, to preventing carcinogenesis, inhibiting the progression of cancers, and improving the efficacy of personal cancer treatment. Gut microbiota are able to play a synergistic effect with chemotherapeutic and immunotherapeutic agents. Based on the studies of gut microbiota, people are also exploring new therapeutic targets, as well as diagnostic, predictive, and prognostic cancer biomarkers using human gut microbiota. The challenging tasks are awaiting. These may include exploring a deeper mechanistic understanding of microbiome in the basic research, accelerating the translation of gut microbiota studies in precision medicine, and finding the way out to human gut microbial biological engineering. Clinical trials using microbiota in combination with chemotherapy or immunotherapy are eagerly expected.

## Competing interests

The authors declare no competing interests.
